# Effect of Grain Size and Surface Roughness on the Normal Coefficient of Restitution of Single Grains

**DOI:** 10.3390/ma13040814

**Published:** 2020-02-11

**Authors:** Chitta Sai Sandeep, Lina Luo, Kostas Senetakis

**Affiliations:** 1 School of Geosciences, Mewbourne College of Earth and Energy, University of Oklahoma, Norman, OK 73069, USA; sschitta2-c@my.cityu.edu.hk or; 2 Department of Architecture and Civil Engineering, City University of Hong Kong, Yeung Kin Man Academic Building, Blue Zone 6/F, Kowloon, Hong Kong, China; linaluo220@gmail.com

**Keywords:** coefficient of restitution, glass balls, granite, rubber, roughness

## Abstract

The coefficient of restitution (COR) represents the fraction of pre-collision kinetic energy remained after the collision between two bodies. The COR parameter plays an important role in the discrete numerical analysis of granular flows or the design of protective barriers to reduce flow energy. This work investigated the COR for grain-block type impacts through comprehensive experiments using a custom-built impact loading apparatus. Glass balls of three different sizes were used as grains. The impact experiments were performed on three different types of materials as base blocks, namely brass, granite and rubber. Experiments on the brass block showed a decrease in COR values with increasing grain size. On the contrary, impacts on granite and rubber blocks showed an increase in COR values with increasing grain size. Additionally, the effect of surface roughness on the COR was investigated. It was revealed that the increase in surface roughness of either the grain or the block reduced the COR values due to the increased plastic deformations of surface asperities.

## 1. Introduction

The collision between particle and block surface is a fundamental problem in solid mechanics, with applications ranging from macroscopic mechanical engineering to microscopic particle technology [1]. A long list of scientific papers used the collision behavior for various applications, for example, sports [2], the temperature dependence of polymer balls [3] and the stability of planetary rings [4]. Additionally, the collision behavior is of interest in the areas of geological and geotechnical engineering research, such as rock falls, sand dunes, volcanic eruptions, landslides and debris flows, seismic hazards and meteoritic impacts [5,6,7,8,9,10,11]. 

The coefficient of restitution (COR) represents the fraction of pre-collision kinetic energy remained after the collision between two bodies. For a perfectly elastic collision with no kinetic energy loss, the value of COR equals 1; and for a perfectly inelastic collision, the value of COR equals 0. Various factors affect the magnitude of kinetic energy loss, such as material type, surface roughness, shape of the colliding bodies, relative velocities. Energy loss is caused by plastic deformations, viscoelastic phenomena or wave propagation (after Seifried et al. [12]). 

Several studies employed the discrete element method (DEM) in the modeling and analysis of various problems, such as the mechanical behavior of ceramics [13], milling processes [14], fractures in concrete [15] and the simulation of granular flows [16]. In DEM, materials are represented as an assembly of spheres which interact according to physical laws. Scholars using DEM analysis generally employ the Hertz–Mindlin nonlinear elastic constitutive law [17] to study particle interactions. However, for a successful application of the Hertz–Mindlin law, researchers need normal and tangential stiffness as well as the interface friction parameters. Recent experimental studies [18,19,20] obtained the values of inter-particle friction (*µ*) at the grain-scale for various materials including glass balls (with an average *µ* ≈ 0.1). The normal stiffness between two bodies can be obtained using quasi-static grain-scale experiments [18,21] or through COR using dynamic impact loading tests [22]. In numerical simulations such as DEM, the normal contact force (*F_N_*) during collisions is calculated as (after Lommen et al. [23], Tang et al. [24]):

(1) $$F_{N}=K_{N}U_{N}+C_{N}\;\frac{dU_{N}}{dt}$$

where *U_N_* is the normal displacement, *K_N_* is the normal contact stiffness, *C_N_* is the damping coefficient of the normal force which depends on the COR. Therefore, it is evident that the parameter COR influences the contact force and thereby the simulation results as well. For example, Yan et al. [25] studied the effects of various factors on DEM results by simulating the discharge of particles from a flat bottom cylindrical container onto a plate. They reported that the repose angle increases with increasing COR values for lower values of *µ,* while the repose angle decreases with increasing COR values for higher values of *µ*. 

A large body of experiments has been carried out to examine the COR of engineered materials. For metallic collisions, Tabor [26] observed that the values of COR are not consistent for materials that undergo plastic deformations. It was reported in that study that COR decreases with increasing impact velocity. Earlier works [27,28] also observed similar trends between COR and impact velocity. Several experimental works were carried out to study the collision behavior of balls colliding on block surfaces [29,30,31,32]. For the collisions between metal-metal or glass-metal impacts, researchers observed a decrease in COR with increasing the size of the impacting ball [33,34]. A previous study by Aryaei et al. [1] also reported a similar trend for metal-metal impacts based on both experimental and analytical evidence. 

In debris flows and other granular flows, grains of different sizes and surface roughness interact and the exchange of momentum at the microscale involves inertial granular collisions, friction between grains, viscous shear and solid-fluid interactions [35]. In the design of barriers for landslides and other granular flows, flexible type barriers or car tire cushioning layers are placed in front of concrete barriers to dissipate energy [36]. Understanding the impact loading behavior of different grain sizes with flexible type materials, such as rubber blocks, helps in designing barriers for energy dissipation. For 3 mm steel balls impacting on conglomerate rock surface, Imre et al. [37] reported an average COR value of 0.87. A recent study by Sandeep et al. [22] reported the impact loading behavior of glass balls, steel balls and quartz sand grains (with a focus on 2 mm size balls) on brass, granite and rubber base blocks. However, compared with metal-metal impacts, a very limited amount of literature is available on the effect of grain size on brittle materials (such as rocks) and rubber blocks.

This paper presents the effect of grain size on the impact loading behavior of different types of materials. Impact experiments were conducted using a custom-built micromechanical loading apparatus and a high-speed photography technique was used to record drop and rebound of the balls. Glass balls of different sizes were dropped on different types of base blocks, namely, brass (metal block), granite (brittle rock) and rubber block. The results are presented in terms of COR emphasizing the properties which are affecting the changes in the impact loading behavior of the ball-block contacts. Additionally, some glass balls were fabricated and made rough to study the effect of surface roughness on the COR so that to provide further insights into the role of surface morphology, at the microscale, on the impact behavior of ball-block contacts. 

## 2. Experimental Apparatus and Materials

### 2.1. Micromechanical Impact Apparatus

**Figure 1:**
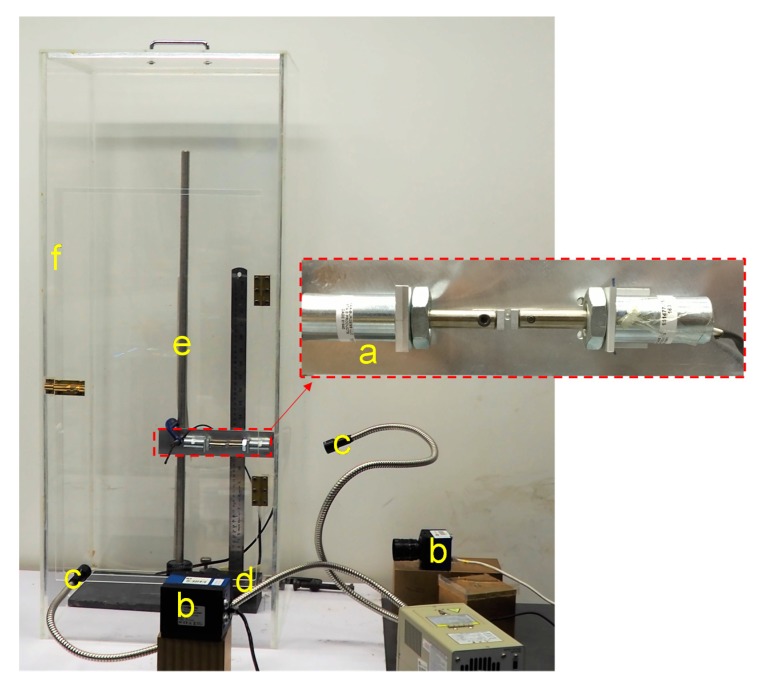
An image of the micromechanical impact apparatus (a) solenoids holding glass ball before the drop test (b) high-speed cameras (c) focused LED’s (d) base plate (e) cantilever pole to adjust height of drop (f) Perspex chamber.

A micromechanical impact apparatus was used to measure the COR of grain and base block impacts for different material combinations. This apparatus was developed inhouse and is described in a recent study by Sandeep et al. [22], but for completeness, it is detailed here as well. An image of the impact loading apparatus is shown in Figure 1. The apparatus consisted of different components as labeled in Figure 1. A system of two solenoids (a) was mounted on a steel frame and they were connected to the cantilever pole (e) which is used to adjust the height of the drop. The grain was initially held between the plungers of the solenoids which were wrapped in the coil springs. The generated electromagnetic energy with the flow of electric current pulls the plungers backward into the solenoids and based on this process, the particle was released on to the base plate (d). The drop and rebound of the particle before and after the impact were captured using high-speed cameras (b) with a shutter speed of one thousand frames per second. The travel path of the grain is focused with LED lights (c) to increase the exposure time without compromising the frame rate. The apparatus is placed into a Perspex chamber (f) to minimize airflow effects. The apparatus can test grains from 1 to 5 mm in diameter and drop heights up to 600 mm can be achieved. 

### 2.2. Materials

In this study, the COR of glass balls in various sizes against base blocks of three different material types was investigated. Commercially available glass balls of three different sizes i.e., 2, 3 and 4 mm in diameter were used as grains. Sandeep and Senetakis [20] obtained the Young’s modulus at the glass ball contacts by performing inter-particle experiments. In order to study the effect of roughness on the impact loading behavior, the surface of several glass balls was fabricated by rubbing them against medium macrogrit sandpaper to increase the roughness. Care was taken to avoid any alteration of the spherical shape of the glass balls while fabricating them. The base blocks used for the impact tests are brass, rubber and granite. The base (blocks) are of 9 × 9 cm square and 6 cm thick. The Poisson’s ratio of the blocks was obtained from literature sources based on the material type [32,38,39,40], while the Young’s modulus of brass and glass balls was adopted based on Marinack et al. [32] and Sandeep and Senetakis [20]. The density of the blocks was estimated in the laboratory. 

The surface roughness of glass balls and base blocks was measured with an optical surface profiler (Veeco NT9300, Bruker, Billerica, MA, USA). For the glass balls and blocks, a field of view of 20 × 20 µm was selected for scanning to determine the approximate roughness. The surface roughness is presented as the root mean square (RMS) roughness denoted as *S_q_* based on Equation (2).

(2) $$S_{q}=\sqrt{\frac{1}{u}\sum_{i=1}^{u}\left(W_{i}^{2}\right)}$$

where *u* = the number of measured data points and *W* = the elevation relative to the base surface. The surface roughness is measured in terms of *S_q_* as this parameter is more sensitive than arithmetic average height for measuring large deviations from the mean line (Gadelmawla et al., 2002).

**Figure 2:**
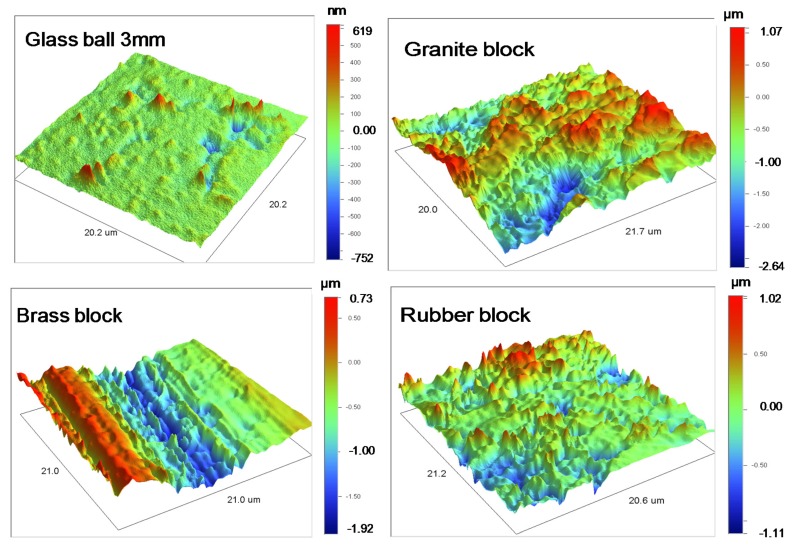
Typical flattened three-dimensional surface topographs of glass ball (3 mm) and base blocks.

For the glass balls, the effect of shape on the surface roughness was removed using a sphere and tilt function present in the Veeco Software (Vision 4.20, Bruker, Billerica, MA, USA). Typical images of flattened (after removing the effect of shape) three-dimensional surface topography of a glass ball and base blocks are shown in Figure 2. For the glass balls, the average surface roughness was estimated based on measurements on ten grains for each different grain size group. For the blocks, the average surface roughness for each block type was estimated based on measurements at ten different locations on their surfaces, which locations were chosen randomly. The average values of surface roughness along with the standard deviation for both the grains and blocks are presented in Table 1. 

## 3. Testing Program and Analytical Expressions

To study the effect of grain size and surface roughness on COR, thirty-three different ball-block combinations were tested including both smooth and rough glass balls of different sizes and different drop heights. Grains were dropped from three different heights of 155, 217, and 300 mm with corresponding impact velocities of 1.74, 2.06, and 2.43 m/s, respectively. At a required height, ten trials were carried out for each glass ball size and base block combination. In total, 290 impact tests were conducted using both smooth and rough glass balls.

**Figure 3:**
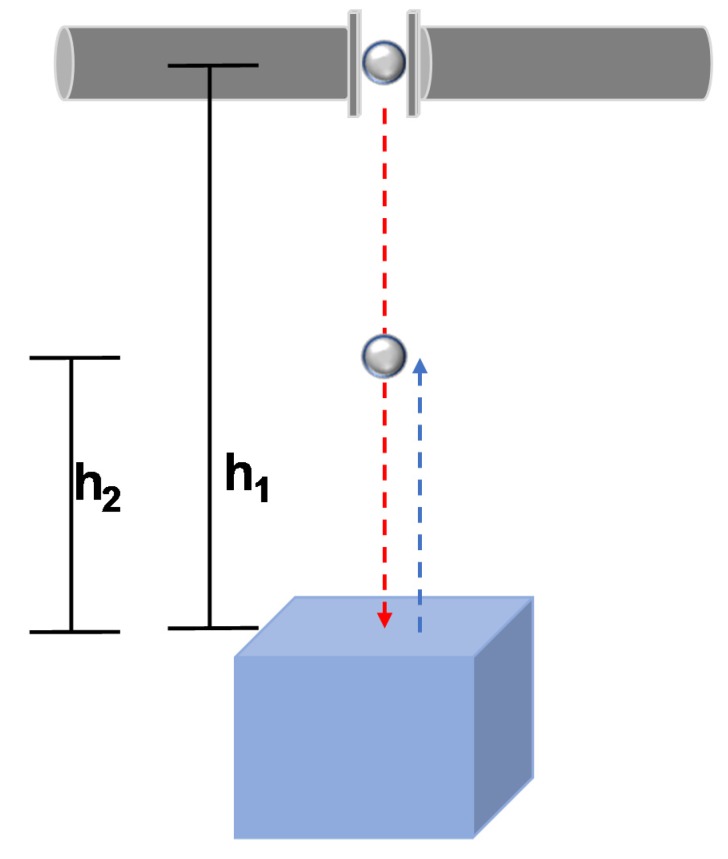
A schematic representation of glass ball impacting on the surface of base block showing initial drop height (h_1_) and rebound height (h_2_).

Figure 3 shows a schematic representation of a ball-block impact. COR is defined as the ratio of relative velocities of the impacting bodies before and after impact as defined in Equation (3):

(3) $$COR=\frac{V_{2b}-V_{2g}}{V_{1g}-V_{1b}}$$

where *V*_1*g*_, *V*_1*b*_ and *V*_2*g*_, *V*_2*b*_ are the velocities of the grain (ball) and the block before and after impact, respectively. In the present study, the base blocks are stationary, therefore the velocity of the blocks reduces to zero. However, obtaining COR by measuring the ball’s velocities can lead to errors even with the minimum possible differences [42]. Therefore, in this study, the COR is obtained using the initial drop height *(h*_1_) and the rebound height (*h*_2_) as presented in Figure 3 and Equation (4):

(4) $$COR=\sqrt{\frac{h_{2}}{h_{1}}}$$

The impact process was recorded by means of high-speed photography and the video was processed to obtain the rebound height. A recent study by Marinack et al. [32] showed that the effect of air drag on the grain velocity (for the range of velocities used in this study) is negligible and the COR values only differ by less than 1%. Repeatability of the tests and data acquisition using the impact loading apparatus used in the present study was presented by Sandeep et al. [22]. 

When two elastic grains of radius *R*_1_ and *R*_2_ are subjected to a concentric force (*F*), the radius of circular contact area (*a*) is proposed by Hertz [43] and it is calculated as presented in Equation (5) as follows:

(5) $$a={\left(\frac{3R^{*}F}{4E^{*}}\right)}^{\frac{1}{3}}$$

where R* and E* are the equivalent radius and Young’s modulus of the grains in contact as given by Equations (6) and (7) as follows:

(6) $$\frac{1}{R^{*}}=\frac{1}{R_{1}}+\frac{1}{R_{2}}$$

(7) $$\frac{1}{E^{*}}=\left[\frac{1-\nu_{1}^{2}}{E_{1}}+\frac{1-\nu_{2}^{2}}{E_{2}}\right]$$

where *ν*_1_ and *E*_1_ are the Poisson’s ratio and Young’s modulus of the first grain and subscript 2 refers to the similar parameters for the second grain. 

For the grain and block contacts, the equivalent radius reduces to Equation (8) and the contact radius is obtained from Equation (9) as presented below:

(8) $$R^{*}=R_{1}$$

(9) $$a={\left(\frac{3R_{1}F}{4E^{*}}\right)}^{\frac{1}{3}}$$

Equation (9) shows that the contact radius for the grain-block contact increases with increasing radius of the grain and decreasing equivalent Young’s modulus. 

Stronge [44] developed a theoretical expression for the COR of elasto-plastic impacts as presented in Equation (10):

(10) $$COR={\left(\frac{V_{y}}{V_{1g}}\right)}^{2}{\left(\frac{8}{5}{\left(\frac{V_{1g}}{V_{y}}\right)}^{2}-\frac{3}{5}\right)}^{0.75}$$

In Equation (10), *V_y_* is the impact velocity needed to initiate yielding. From theoretical observations (Equation (10)), grain size has no effect on COR. However, as highlighted in the earlier sections, experimental studies published in the literature [28,30] which were conducted on metal surfaces observed a decrease in COR with the increase in grain size. 

## 4. Results and Discussions

### 4.1. Effect of Size on COR

**Figure 4:**
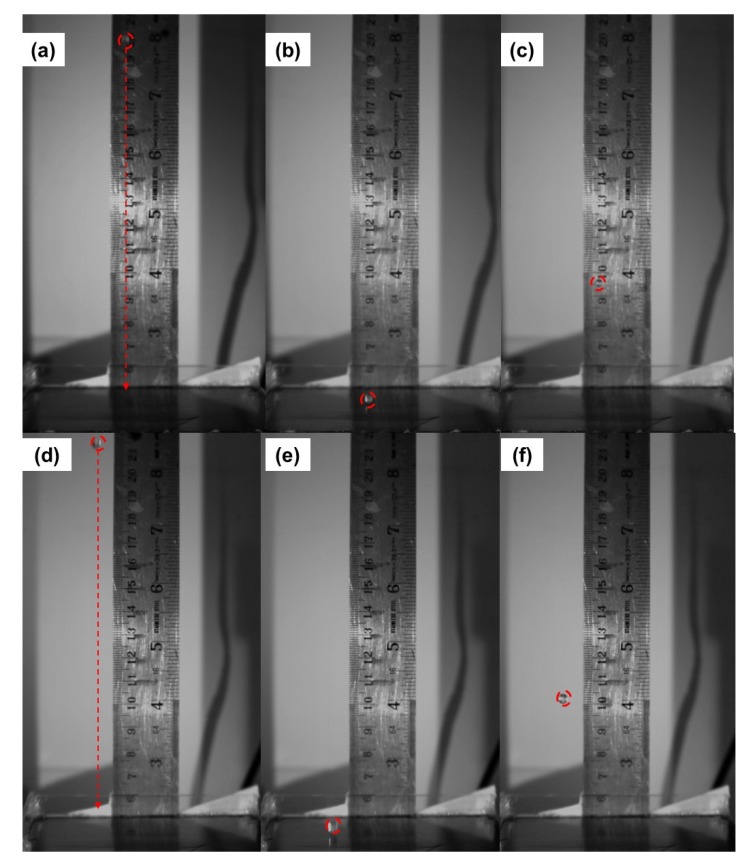
Various stages of glass ball impacting on rubber block (**a**–**c**) 3 mm glass ball (**d**–**f**) 4 mm glass ball.

Figure 4 shows various stages of 3 and 4 mm sized glass balls impacting on the surface of the rubber block at an impact velocity of 2.43 m/s. The glass balls rebounded following the same initial drop path. From Figure 4, it is observed that the rebound height of 4 mm sized glass balls is greater than that of 3 mm. Table 2 presents a summary of the average values of COR for the impacts of glass balls with various base block combinations at different impact velocities. The minimum and maximum absolute values of the standard deviation in COR values for smooth glass ball impacts on all the base block combinations presented in Table 2 are 0.004 and 0.013, respectively. The range of the observed scatter is very low in magnitude for glass balls due to their spherical shape and consistent morphological and elastic characteristics. For natural quartz sand grains, Sandeep et al. [22] observed higher values of the standard deviation which they attributed to irregular shape and defects in the grains compared with engineered materials such as glass and chrome steel balls. 

**Figure 5:**
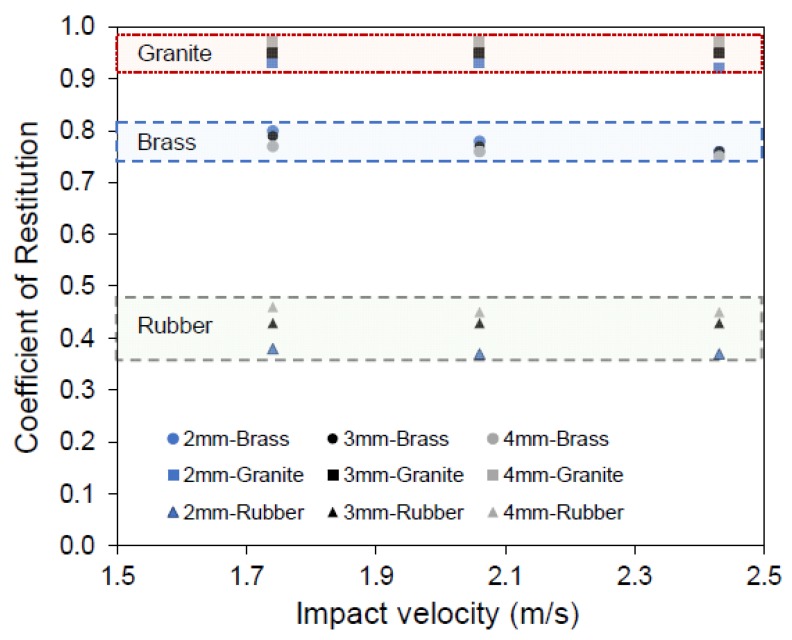
The average values of the coefficient of restitution for 2, 3 and 4 mm sized glass balls impacting on various base blocks.

Figure 5 shows the variation of COR with impact velocity for glass balls of different sizes impacting on various base blocks. In the legends of Figure 5 and Figure 7, the grain-block combinations are specified with grain size in the first position and base block in the second position. For example, in the legend of Figure 5, 2 mm-Brass denotes the grain size to be 2 mm glass ball and the base block to be brass. From Table 2 and Figure 5, it is observed that the COR value decreases with increasing grain size and impact velocity for the impacts on the brass block. For example, the average COR values of 2 and 4 mm sized glass balls impacting on the brass block at an impact velocity 1.74 m/s are 0.80 and 0.77, respectively. Earlier works by Aryaei et al. [1] and Marinack et al. [32] observed similar behavior of drop-in COR values with the increase in grain size for the impacts on metal surfaces. Aryaei et al. [1] attributed the drop in COR value for the large size grains to an increase in the contact area (Equation (8)) and higher effective plastic deformation for the impacts on metal surfaces. However, the rate of reduction in COR with the increase in impact velocity reduced with the increase in grain size. For example, with the change in impact velocity from 1.74 to 2.43 m/s, average COR values reduced about 5% and 2% for 2 and 4 mm glass balls impacting on the brass block.

Contrary to the impacts on the metal surfaces, i.e., brass blocks in this study, the impacts on granite and rubber blocks showed an increasing trend of COR values with increasing grain size. For the impacts on the brass block, notable plastic deformations were observed visually on the brass surface after the tests. However, for the impacts on rubber and granite blocks, no plastic deformation was observed post-collision visually. Additionally, there is no significant influence of the impact velocity on the COR for the impacts on granite and rubber blocks. For the impacts on granite and rubber blocks, COR values reduced by 0–2% only with the increase in impact velocity from 1.74 to 2.43 m/s. For impacts of 3 mm steel balls on conglomerate rock specimen, Imre et al. [37] reported a drop of 0.5% in the COR values with increasing impact velocity from 1.7 to 1.9 m/s. However, a very limited amount of data published in the literature is available on the variation of COR with grain size for the impacts on rock and rubber blocks. 

In this study, it is observed that the average COR values increased about 4–5% and 18–20% with the increase in grain size from 2 to 4 mm for the impacts on granite and rubber blocks, respectively. Equations (9) and (10) imply that the contact area increases with increasing grain size and the COR is not influenced by grain size. However, for the tests on different material types in this study, it is clear that the COR is affected by the grain size and material type as well and that the change of COR with grain size becomes more important when the grains collide on softer blocks. This may also have implications in numerical analyses, as the COR of impacted grains on barriers and the way COR changes with the size of grains is dependent on the type of barriers used. 

It is worth noticing that the impact energy increases with the increase in grain size. Granite blocks are brittle under low pressures, which is the case in the current study and absorb relatively less energy prior to fracture. On the contrary, the brass blocks are ductile in nature and deform plastically during the impacts, so that they absorb a greater amount of energy. Therefore, with the increase in grain size, COR values increased for the impacts on granite and decreased for the impacts on brass. Similarly, for the rubber blocks with no plastic deformations, the COR values increased significantly with increasing grain size. Researchers using DEM in the analysis of granular flows or impacts on rubber type barriers should account for these changes in COR with grain size for a better understanding of granular flow behavior. 

### 4.2. Effect of Surface Roughness on COR

In order to study the effect of surface roughness on COR values, an additional set of tests was performed by impacting 3 mm sized rough glass balls (S*_q_* = 387 nm) on various base blocks at two different impact velocities and the results are summarized in Table 2. Ten trials were performed on rough glass balls at each impact velocity and base block combination. The minimum and maximum absolute values of the standard deviation in the COR values for rough GB impacts on all base block combinations presented in Table 2 are 0.009 and 0.021, respectively. The observed standard deviations for the impacts of rough GB are greater than the smooth GB, this is possibly due to the greater magnitude of standard deviation in roughness for the rough GB used in this study. To check the accuracy of the reported values, additional experiments (additional to those reported in Table 2) were conducted by impacting smooth and rough glass balls (3 mm in diameter) on brass surface at an impact velocity of 1.74 m/s. Ten trials were conducted in each additional case and the values of COR is 0.78 and 0.75 for the impacts of smooth and rough glass balls, respectively. Within the observed standard deviations, these values are similar to the values reported in Table 2.

In the recent study by Sandeep et al. [22] on rough quartz sand grains, oblique rebound of grains was reported due to their irregular shapes and surface morphology. For these impacts, they used the method proposed by Banks et al. [45] and Wang et al. [46] to measure the COR. However, in this study on rough glass balls no noticeable oblique behavior was observed through the high-speed cameras which are placed perpendicular to each other (Figure 1). Greater magnitude of oblique rebound angles during collisions are most likely to be the result of morphology influence in terms of particle shape rather than roughness (i.e., meso-scale morphology expressed by particle shape is more crucial compared with micro-to-nano scale morphology expressed with roughness to cause no vertical rebound of the grains). However, oblique rebound angles can also be observed if the experiments were conducted at an impact angle (for example: Sommerfeld and Huber [47], Cruger et al. [48], Tang et al. [24]).

**Figure 6:**
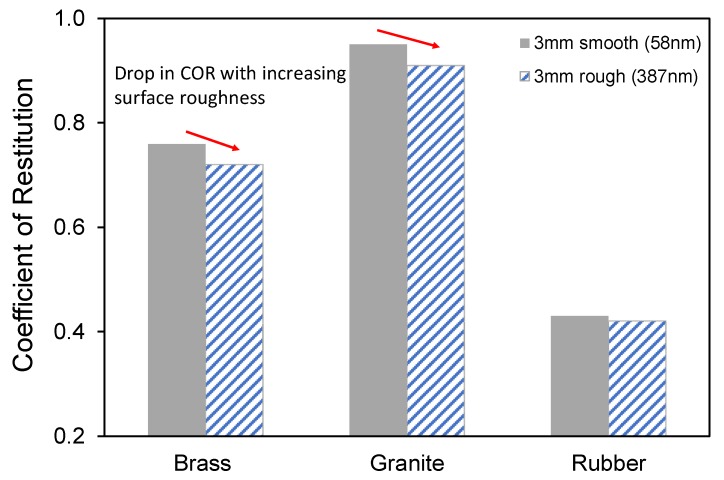
Variation of the coefficient of restitution with surface roughness of 3 mm in diameter glass balls (impact velocity = 2.43 m/s).

Histograms in Figure 6 show the variation of COR values for smooth and rough glass balls impacting on various base blocks at an impact velocity of 2.43 m/s. From Figure 6 and Table 2, it is observed that the COR values generally decrease with increasing surface roughness of the glass ball for the impacts on brass and granite blocks. At an impact velocity of 1.74 m/s, increase in surface roughness of the glass ball reduced the average value of COR about 6% and 8% for the collisions on brass and granite blocks, respectively. For the impacts on rubber blocks, no effective changes were observed in the COR values with increasing surface roughness of the glass balls. This is possibly due to the very low Young’s modulus of the rubber block. 

**Figure 7:**
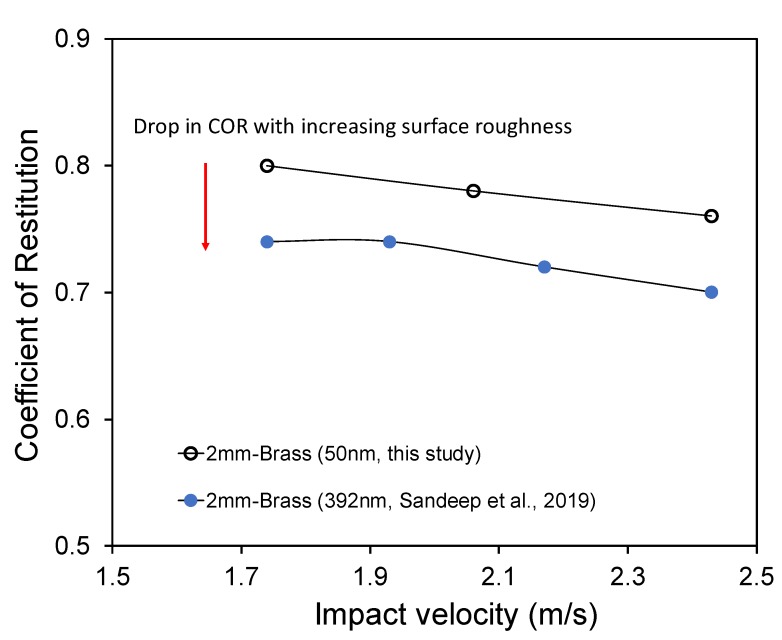
Variation of the coefficient of restitution with surface roughness of brass block.

Figure 7 shows the COR values for 2 mm in diameter glass balls impacting on brass blocks of two different surface roughness values (S*_q_* = 50 nm, present study; S*_q_* = 392 nm, after [21]). From Figure 7, it is observed that the COR value decreases with increasing surface roughness of the base block as well. From Figure 6 and Figure 7, it is understood that the COR values are sensitive to changes in surface roughness of both the grain and the base block. For small grain sizes, even low velocity impacts can cause high stresses enough to trigger plastic deformations of the asperities. For the current experiments, the maximum possible impact load for a 3 mm grain is less than 0.5 N (after Zhang and Vu-Quoc [49], Aryaei et al. [1]). For low velocity impacts (0.3–0.6 m/s) of soft polystyrene particles on smooth and rough steel surfaces, Krull et al. [50] reported a decrease of COR for the impacts on rough surfaces which they attributed to the possible plastic deformation of polystyrene spheres. For the impacts with low impact angles, they also observed minute rebound angle (2–3°) for both the smooth and rough surface impacts. 

In grain-grain static normal loading experiments for several material combinations (approximate grain size is 2–3 mm), Sandeep and Senetakis [20,51] reported plastic deformation of asperities at a very low magnitude of normal forces (even less than 0.1 N) which mainly depends on material type and surface roughness. They reported a greater magnitude of plastic deformation for rough surfaces. Therefore, the current dynamic impact loading tests can also result in a greater plastic deformation for rough surfaces which leads to reduced COR values. 

**Figure 8:**
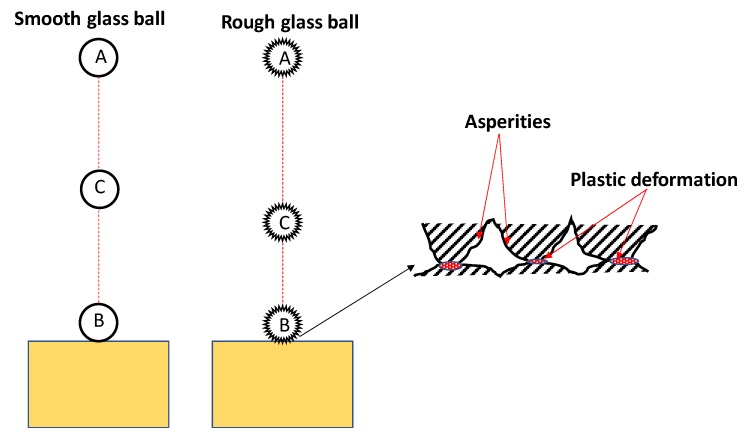
A schematic representation of smooth and rough glass ball impacts on base block (A) initial position (B) during impact (C) position after rebound.

A schematic representation of the smooth and rough glass balls impacting on base blocks is presented in Figure 8 which shows plastic deformation of rough asperities during impacts. The decrease in COR values with the increase in surface roughness is possibly due to the additional loss of kinetic energy through plastic deformations of micro-asperities during impact. Granular flows like debris flows and landslides consist, predominantly, of various sized grains and water mixtures [35]. However, limited research has been devoted on the impact loading behavior of natural geological grains. In the next research cycle, we plan to investigate the role of grain size and humidity on the COR of geological-natural materials. 

## 5. Conclusions

The effect of grain size and material type on COR is investigated by conducting experiments using glass balls of different sizes impacting blocks of three different material types. High-speed photography technique was used to obtain the rebound height and the value of COR was calculated based on analytical expressions. Additionally, the effect of surface roughness on COR was also investigated by conducting tests using glass balls of different surface roughness. The conclusions could be summarized as follows:

(1) For the impacts on brass blocks, the COR decreases with increasing grain size. Materials with dominantly ductile behavior, such as metals, are prone to plastic deformations upon impacts which majorly contribute to the energy losses. 

(2) Impacts on granite and rubber blocks showed an increasing trend in COR with increasing grain size. For materials which do not exhibit plastic deformations upon impact such as rubber blocks, the value of COR increased by 20% with increasing grain size from 2 to 4 mm. 

(3) For collisions with brass and granite blocks, an increase in surface roughness either for the block or grain resulted in a decrease of COR. The additional energy loss is due to plastic deformations of the surface micro-asperities. 

(4) For glass grain-rubber block impacts, no noticeable changes were observed in the COR values with increasing surface roughness of the grains. This is due to the very low Young’s modulus of the rubber blocks. 

To the best knowledge of the authors, this is the first study to collectively compare the effect of grain size and surface roughness on the collision behavior of glass balls with different material types such as, granite, brass and rubber blocks. The results stemming from the present work may provide a basis to calibrate the models used in DEM platforms. Researchers using discrete type numerical modeling techniques should account for material type, grain size and surface roughness while simulating problems related to collisions, such as granular flows and impacts on rubber barriers.

## Figures and Tables

**Table 1:** Summary of material properties for the grains and blocks used in the impact tests.

Type	Material	Density (t/m^3^)	Poisson’s Ratio	Surface Roughness (nm)	E (GPa)
Block	Brass (N2)	8.50	0.35 ^β^	50 *	100 ^β^
	Granite	3.00	0.30 ^Λ^	412 ± 35	86
	Rubber	1.50	0.50 ^ψ^	270 ± 42	0.1
Grain	Glass (smooth) (2 mm)	2.60	0.30 ^#^	95 ± 28	58 *
	Glass (smooth) (3 mm)			58 ± 18	
	Glass (smooth) (4 mm)			67 ± 23	
	Glass (rough) (3 mm)			387 ± 81	-

^#^ Gu and Yang [41], ^β^ Marinack et al. [32], ^Λ^ Gupta and Rao [39], ^ψ^ Holownia [40], * Sandeep and Senetakis [20].

**Table 2:** Average value of coefficient of restitution for 2, 3 and 4 mm sized glass balls with various block combinations at different impact heights.

Block	Surface Condition of Grain	Grain Size (mm)	Coefficient of Restitution Values		
			h_1_ (mm): 155	217	300
			Vel (m/s): 1.74	2.06	2.43
Brass	Smooth	2	0.80	0.78	0.76
		3	0.79	0.77	0.76
		4	0.77	0.76	0.75
	Rough	3	0.74	-	0.72
Granite	Smooth	2	0.93 *	0.93	0.92 *
		3	0.95	0.95	0.95
		4	0.97	0.97	0.97
	Rough	3	0.87	-	0.91
Rubber	Smooth	2	0.38 *	0.37	0.37 *
		3	0.43	0.43	0.43
		4	0.46	0.45	0.45
	Rough	3	0.41	-	0.42

* Data after Sandeep et al. [22].
